# AP2 transcription factor CsAIL6 negatively regulates citric acid accumulation in citrus fruits by interacting with a WD40 protein CsAN11

**DOI:** 10.1093/hr/uhaf002

**Published:** 2025-01-06

**Authors:** Han Han, Yu-Jia Li, Shariq Mahmood Alam, Tian Zhou, Muhammad Abbas Khan, Aye Myat Thu, Yong-Zhong Liu

**Affiliations:** National Key Laboratory for Germplasm Innovation & Utilization of Horticultural Crops, College of Horticulture & Forestry Sciences, Huazhong Agricultural University, No.1 Shizishan Street, Hongshan District, Wuhan 430070, China; National Key Laboratory for Germplasm Innovation & Utilization of Horticultural Crops, College of Horticulture & Forestry Sciences, Huazhong Agricultural University, No.1 Shizishan Street, Hongshan District, Wuhan 430070, China; National Key Laboratory for Germplasm Innovation & Utilization of Horticultural Crops, College of Horticulture & Forestry Sciences, Huazhong Agricultural University, No.1 Shizishan Street, Hongshan District, Wuhan 430070, China; National Key Laboratory for Germplasm Innovation & Utilization of Horticultural Crops, College of Horticulture & Forestry Sciences, Huazhong Agricultural University, No.1 Shizishan Street, Hongshan District, Wuhan 430070, China; National Key Laboratory for Germplasm Innovation & Utilization of Horticultural Crops, College of Horticulture & Forestry Sciences, Huazhong Agricultural University, No.1 Shizishan Street, Hongshan District, Wuhan 430070, China; National Key Laboratory for Germplasm Innovation & Utilization of Horticultural Crops, College of Horticulture & Forestry Sciences, Huazhong Agricultural University, No.1 Shizishan Street, Hongshan District, Wuhan 430070, China; National Key Laboratory for Germplasm Innovation & Utilization of Horticultural Crops, College of Horticulture & Forestry Sciences, Huazhong Agricultural University, No.1 Shizishan Street, Hongshan District, Wuhan 430070, China

## Abstract

Citric acid accumulation is an essential process in citrus fruits that determines fruit flavor and marketability. The MBW complex transcription factor genes, *CsAN11*, *CsAN1*, and *CsPH4* play key roles in regulating citric acid accumulation. Although how to regulate *CsAN1* or *CsPH4* was widely investigated, studies on the regulation of *CsAN11* are scarce. In this study, we characterized the AP2/ERF (APETALA2/ethylene response factor) transcription factor gene *CsAIL6*, which is lowly expressed in high-acid citrus varieties and highly expressed in low-acid citrus varieties. Overexpressing *CsAIL6* obviously decreased the citric acid content in citrus fruits, calli, or tomatoes, whereas silencing *CsAIL6* significantly increased the fruit citric acid content. Additionally, transcript levels of *CsAN11*, *CsAN1*, and *CsPH4* were significantly increased by silencing *CsAIL6*; only the *CsAN11* transcript level was significantly decreased by overexpressing *CsAIL6*. Similarly, only the tomato *AN11* (*SIAN11*) transcript level in *CsAIL6* stably overexpressing fruits was markedly lower than that in wild-type (WT) fruits. Further experiments revealed that overexpressing *CsAN11* significantly increased the organic acid content but had no obvious influence on the *CsAIL6* transcript level; in addition, CsAIL6 only interacts with CsAN11, rather than with CsAN1 and CsPH4 of the MBW complex. Taken together, our findings verified that CsAIL6 negatively regulates citric acid accumulation through directly interacting with the WD40 protein CsAN11, which provides a new mechanism for citric acid accumulation in fruits through the regulation of the MBW complex.

## Introduction

As a primary metabolite, organic acid content and composition play essential roles in maintaining fruit flavor and quality [1]. Citric acid is the predominant organic acid in most varieties of citrus fruit and has significant influences on fruit organoleptic traits and market acceptability [2, 3]. The difference in citric acid accumulation among citrus varieties imparts unique flavors to citrus fruits, resulting in different consumer preferences [4, 5]. Citric acid is also involved in various metabolic processes such as photosynthesis, respiration, and amino acid, aromatic, ester, and phenolic biosynthesis [2, 6, 7]. In the flower petals of some plants, high level of acidity leads to blue petals [8]; moreover, higher citric acid level can delay citrus fruit postharvest senescence processes by providing more adenosine triphosphate (ATP) and intermediate metabolites by consuming citric acid, inhibiting lignification, improving osmoregulation, and inducing antioxidant defense systems [9–13]. Thus, citric acid plays multiple roles in citrus fruits, and comprehensively elucidating the mechanism of citric acid accumulation is crucial for improving citrus fruit quality.

Citric acid accumulation is a pivotal biochemical process and is intricately regulated by biosynthesis, degradation, and vacuole storage [2]. It is an intermediate in the tricarboxylic acid cycle and is formed by citrate synthase (CS) catalyzing the condensation of acetyl-CoA and oxaloacetate in mitochondria. Then, it can be further utilized by ATP-citrate lyase (ACL) or aconitase (ACO). On the other hand, citric acid can also be transported via vacuolar proton pumps into the cell vacuole for storage [4].

To date, several studies showed that change in CS activity is positively correlated with citric acid content [14, 15]. Nevertheless, more evidence has confirmed that CS activity has no significant correlation with citric acid content [16–18]. Similarly, ACO has been shown to affect citric acid accumulation in fruit [19–21]. Guo *et al.* [22] suggested that ACO activity negatively controls citric acid content and that the inhibition of ACO leads to an increase in citric acid content. However, Huang *et al.* [23] reported no significant correlation between acidity and ACO gene expression in low-acid citrus and suggested that ACO may not be the key factor determining the diversity of citrus fruit acidity. For storage, the transport of citric acid across the tonoplast to be stored in the vacuole is regulated by proton pumps, which include vacuolar ATPase (V-ATPase), vacuolar PPase (V-PPase), and P-type ATPase [24]. For example, V-ATPase plays a pivotal role in lemon hyperacidification [25], whereas Kriedel *et al.* [26] reported that vacuole acidification may depend on both V-ATPase and V-PPase. Moreover, a P-type ATPase demonstrated its role in regulating citrus fruit vacuolar pH and organic acid accumulation since *AtAHA10* and *PhPH5* were reported to regulate vacuolar acidification in *Arabidopsis* [27] and petunia [8], respectively. In detail, Aprile *et al.* [28] initially revealed that a citrus P-ATPase gene (homolog of *AHA10*) was highly expressed in sour lemon but not in sweet lemon. Shi *et al.* [29] subsequently identified a *PhPH5* homolog gene (*CsPH8*) in citrus and reported that its lower transcript level was the main cause for lower citric acid accumulation in acidless citrus cultivars (‘Honganliu’ and ‘Wusuan’ pomelo). Further studies indicated that the citrus P_3A_-ATPase proton pump gene (*CitPH5* or *CsPH8*) is the key factor that diversifies citric acid contents in citrus fruits [30, 31].

The regulatory mechanism of the P-type proton pump gene was subsequently extensively investigated. Particularly, the MBW complex, formed by the interaction of an MYB transcription factor (PH4), basic helix–loop–helix (AN1), and WD40 proteins (AN11), contributes to vacuolar acidification by influencing the transcript level of the p-type proton pump gene in *Arabidopsis* [32], petunia [33], and grapevine [34]. In citrus, the MBW complex (CitAN1–CitPH4–CitAN11) also exists and is involved in regulating the citrus P_3A_-ATPase proton pump gene transcript for vacuolar acidification [31]. Furthermore, the regulation of the three MBW components was also investigated. CitPH4 was considered to play a pivotal role in citric acid accumulation of citrus fruits because of the numerous variations in sequence and expression among citrus-related genera [31, 35]; AN1 (Noemi/bHLH) can be interacted by PH4 (MYB) and AN2 (Ruby1/MYB) and collectively affect the contents of anthocyanins, proanthocyanidins, as well as citric acid in fruits [36–38]. Moreover, the transcription factor CitTRL (citrus TRIPTYCHON-LIKE) was found to negatively regulate citric acid content by directly interacting with *CitAN1* [5]. Additionally, CsABF3 (ABA-responsive element binding factor 3) promoted citric acid accumulation under drought stress directly through the upregulation of *CsAN1* or *CitAN1* transcript level [39]. However, functional knowledge regarding the regulation of citrus *AN11* for citric acid accumulation is still scarce.

AP2/ERF (APETALA2/ethylene response factor) transcription factors are widely present in plants and regulate fruit development and flavor [4, 40–42]. Here, we identified the AP2 transcription factor gene *CsAIL6* in citrus. The present study demonstrated that CsAIL6 is the key factor that interacts with CsAN11 to modulate citric acid accumulation in citrus fruits. This finding provides another molecular mechanism for citric acid accumulation through regulating the MBW complex, which will contribute to the improvement of fruit flavor through genetic engineering or developing cultivation measures in the future.

## Results

### Identification and characterization of *CsAIL6* in citrus

**Figure 1:**
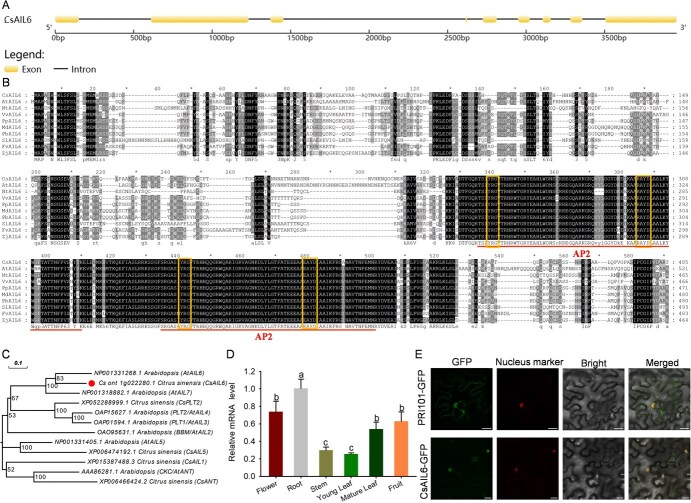
Bioinformatics, expression, and subcellular localization analyses of CsAIL6. **A)** Schematic gene structure of *CsAIL6* in citrus. **B)** Multiple protein sequence alignment of CsAIL6 and its homologous sequences. The lines above the sequence indicate the AP2 domains. The box indicates the conserved YRG and RAYD regions. At, *Arabidopsis thaliana*; Fv, *Fragaria vesca*; Md, *Malus domestica*; Nt, *Nicotiana tabacum*; Pb, *Pyrus bretschneideri*; Pp, *Pyrus persica*; Sl, *Solanum lycopersicum*; Vv, *Vitis vinifera*; Zj, *Ziziphus jujube*; Cs, *Citrus sinensis.*  **C)** Phylogenetic tree of CsAILs and AtAILs. CsAIL6 is highlighted with a solid circle. The phylogenetic tree was constructed using the NJ bootstrap method (1000 replicates) after sequence alignment with MEGA-X (v10.2.2) software. **D)**  *CsAIL6* expression levels in different ‘Newhall’ navel orange tissues. Different lowercase letters indicate statistically significant differences by the Duncan test (*P* < 0.05). **E)** Subcellular localization of the CsAIL6-GFP fusion protein in the epidermal cells of *N. benthamiana* leaves. Scale bar = 25 μm.

We isolated the full-length sequence of *CsAIL6* from ‘AL’ sweet orange, and found that its identity was >99.0% with the sequence of Cs_ont_1g022280.1 in the citrus reference genome (Citrus Pan-genome to Breeding Database, http://citrus.hzau.edu.cn/index.php). Structural analysis revealed that the length of *CsAIL6* coding sequence is 1611 bp and contains eight exons and seven introns (Fig. 1a). Amino acid sequence alignment revealed that CsAIL6 includes two typical highly conserved AP2 DNA-binding domains with YRG and RAYD regions (Fig. 1b). Phylogenetic analysis revealed that CsAIL6 was grouped with the *Arabidopsis thaliana* AIL6 (AtAIL6) (Fig. 1c). Furthermore, the transcript level of *CsAIL6* could be examined in different citrus tissues. Specially, *CsAIL6* was highest expression in fibrous roots, followed by flowers and fruits (Fig. 1d). Subcellular localization analysis revealed that the CsAIL6-GFP fusion protein was detected exclusively in the nucleus and completely merged with the mCherry fluorescence of the nuclear marker (Fig. 1e). In addition, we analyzed the transcriptional activation activity of CsAIL6, and the results showed that yeast cells expressing BD-CsAIL6 could not grow well on SD-Ade/-His/−Trp plates with X-α-gal (Supplemental Fig. S1).

### *CsAIL6* transcript profiles and acidity in fruits of different citrus cultivars

**Figure 2:**
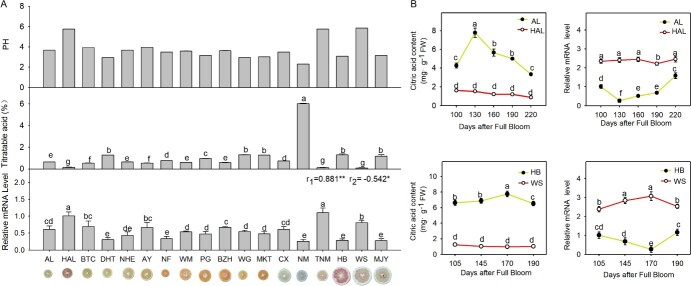
Dynamic profiles of *CsAIL6* transcript levels and citric acid contents during fruit development in different citrus varieties. **A)** Correlation analysis of *CsAIL6* transcript levels with TA (%) contents and pH values in fruits from different citrus varieties. **B)**  *CsAIL6* expression profiles and citric acid contents of different citrus varieties during different developmental stages. FW: fresh weight; AL: ‘Anliu’ sweet orange; HAL: ‘Hong Anliu’ sweet orange; HB: ‘Hirda Buntan’ pomelo; WS: ‘Wusuan’ pomelo. r_1_ indicates the Pearson correlation coefficients between the *CsAIL6* transcript levels and the pH values of 18 citrus varieties, and r_2_ indicates the Pearson correlation coefficients between the *CsAIL6* transcript levels and the TA contents of 18 citrus varieties. Different lowercase letters indicate statistically significant differences by the Duncan test (*P* < 0.05). The asterisks indicate a significant correlation (*P < 0.05; **P < 0.01). The error bars indicate the standard deviation of three replicates (±SD).

Eighteen citrus cultivars with different acidity (Supplemental Table S1) were selected to investigate the relationships between fruit pH or titratable acid (TA) content and the *CsAIL6* transcript level. *CsAIL6* was highly expressed in ‘HAL’, ‘TNM’, and ‘WS’ fruits with low TA, whereas its expression levels were lower in ‘AL’, ‘NM’, and ‘HB’ fruits with high TA. Moreover, the *CsAIL6* expression level in fruits was correlated significantly and positively with pH (r_1_ = 0.881) and correlated significantly and negatively with the TA content (r_2_ = −0.542) (Fig. 2a).

In addition, five development stages [100, 130, 160, 190, and 220 days after full bloom (DAFB)] of ‘AL’ and ‘HAL’ fruits and four development stages (105, 145, 170, and 190 DAFB) of ‘HB’ and ‘WS’ fruits were sampled. During fruit development, the citric acid contents in ‘HAL’ and ‘WS’ fruits were markedly lower than those in ‘AL’ and ‘HB’ fruits, whereas *CsAIL6* expression were obviously higher in ‘HAL’ and ‘WS’ fruits than in ‘AL’ and ‘HB’ fruits. Especially, the changes in the *CsAIL6* transcript levels throughout fruit development were almost opposite to the changes of the citric acid content (Fig. 2b). These findings showed that the citric acid content is strongly and negatively associated with *CsAIL6* expression.

### Functional confirmation of CsAIL6 in transgenic citrus fruits and tomato plants

**Figure 3:**
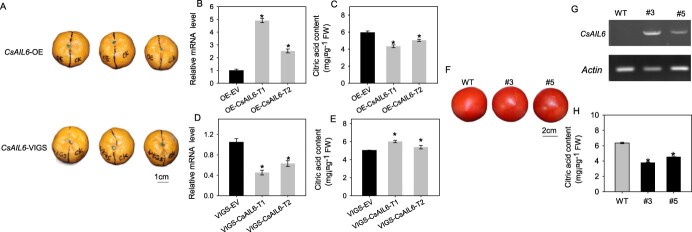
Functional characterization of *CsAIL6* in transgenic citrus fruits and tomato lines. **A)** Transient OE and VIGS of *CsAIL6* in citrus fruits. EV, empty vector. **B**, **D)** Relative expression levels of *CsAIL6* in transgenic citrus fruits. **C**, **E)** Citric acid contents in citrus fruits. **F)** Phenotypes of mature tomato fruits of the WT and OE transgenic lines (#3 and #5). **G)** Analysis of *CsAIL6* expression in transgenic and WT tomato fruits by RT-PCR. **H)** Citric acid contents in transgenic and WT tomato fruits. Asterisks (*) indicate significant differences determined by the *t*-test (*P* < 0.05). The error bars indicate the standard deviation of three replicates (±SD). FW, fresh weight. WT, wild-type.

To verify the function of CsAIL6, overexpression (*CsAIL6*-OE) and virus-induced gene silencing (*CsAIL6*-VIGS) vectors were constructed and transiently transformed into citrus fruits (Fig. 3a). It was found that the *CsAIL6* transcript level was significantly higher in the OE fruits than in the wild-type (WT) fruits (Fig. 3b). Conversely, the citric acid content was significantly lower in the OE fruits than in the WT fruits (Fig. 3c). Moreover, the *CsAIL6* transcript level was significantly lower and the citric acid content was significantly higher in the silenced lines than in the WT lines (Fig. 3d and e).

Furthermore, we overexpressed *CsAIL6* in ‘Micro-Tom’ tomato plants. Two stable *CsAIL6* OE lines (#3 and #5) of the T2 generation were obtained and confirmed by real-time polymerase chain reaction (RT-PCR) analysis (Fig. 3f–g). The citric acid contents of the mature fruits were significantly lower than those of the WT fruits (Fig. 3h). These results further confirmed that *CsAIL6* negatively regulates citric acid accumulation in citrus fruits.

### Transcript analysis of genes related to citric acid accumulation in the transgenic lines

**Figure 4:**
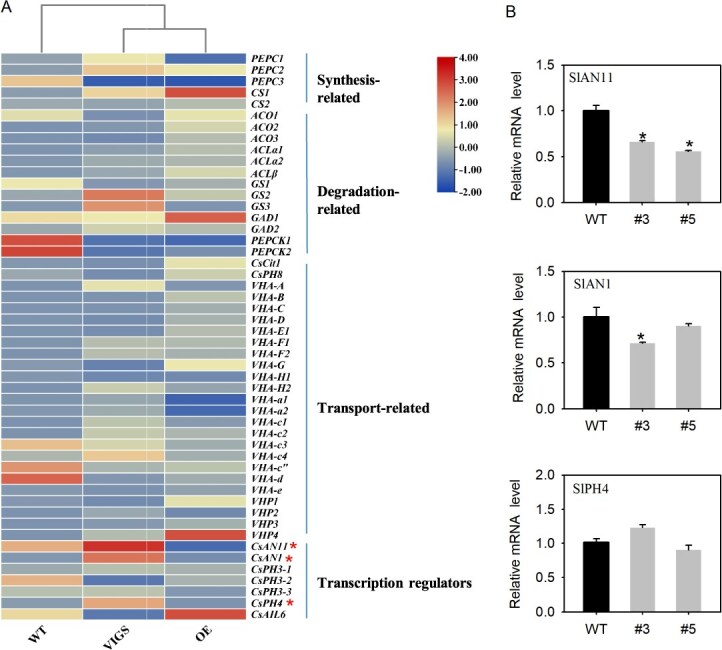
Expression levels of genes involved in citric acid accumulation in transgenic citrus and tomato lines. **A)** Heat map of citric acid accumulation-related gene expression levels in WT, transient OE, and VIGS of *CsAIL6* citrus fruits. **B)** Relative expression levels of *SlAN11*, *SlAN*1, and *SlPH4* in transgenic and WT tomato fruits. Asterisks (*) indicate significant differences determined by the *t*-test (*P* < 0.05). The error bars indicate the standard deviation of three replicates (±SD).

By using *CsAIL6* transgenic lines, we measured the transcript levels of 50 genes related to citric acid synthesis, degradation, transport, and transcription regulators in the fruits of the WT, *CsAIL6-*overexpressing, and *CsAIL6-*silenced lines (Fig. 4a). Quantitative RT-PCR (qRT-PCR) analysis showed that some genes were differently expressed in VIGS and OE fruits compared to the control fruits; e.g. *PEPC1* transcript level was upregulated and downregulated while transcript levels of *ACO*, *ACL, CsCit1,* and *CsPH8* were downregulated and upregulated in VIGS and OE fruits, respectively; however, the difference in expression level of these genes was indistinctive between VIGS or OE fruits and control fruits. Strikingly, the relative mRNA levels of *CsAN11*, *CsAN1*, and *CsPH4* were obviously higher in the *CsAIL6-*VIGS lines than in the WT lines, and only the relative mRNA level of *CsAN11* was notably lower in the *CsAIL6*-OE lines than in the WT lines. We further measured the relative mRNA levels of *SlAN11*, *SlAN1*, and *SlPH4* in WT and transgenic tomato lines. It was found that the expression level of *SlAN11* was observably lower only in the fruits of the transgenic tomato plants than in the WT lines (Fig. 4b). These results demonstrated that *CsAIL6* negatively regulates *CsAN11* expression.

### Interaction analysis of CsAIL6 and MBW complex components

**Figure 5:**
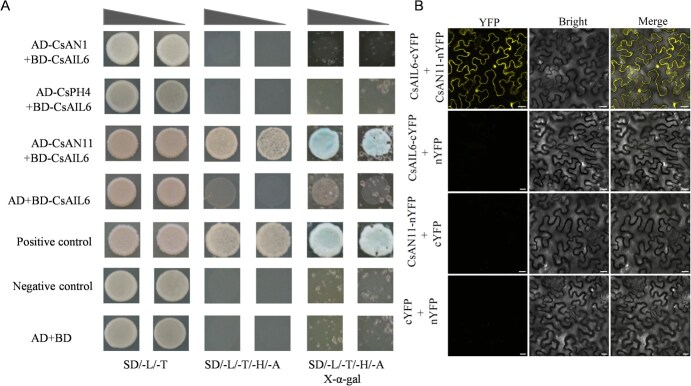
The CsAIL6 protein interacts with the CsAN11 protein. **A)** Y2H assays confirmed the interaction between CsAIL6 and CsAN11. **B)** The interaction between CsAIL6 and CsAN11 was also confirmed by BIFC. CsAIL6-cYFP and CsAN11-nYFP interacted to form a functional YFP in *N. benthamiana* cells. CsAIL6-cYFP + nYFP, CsAN11-nYFP + cYFP, and cYFP + nYFP were used as negative controls. Scale bar =25 μm.

The interaction between CsAIL6 and the MBW complex components was further assessed by a yeast two-hybrid (Y2H) assay. Interestingly, there was an interaction between CsAIL6 and CsAN11, whereas interaction was not detected between CsAIL6 and CsAN1 or CsPH4 (Fig. 5a). Subsequently, bimolecular fluorescence complementation (BIFC) analysis was conducted to confirm the relationship between CsAIL6 and CsAN11. Finally, the yellow fluorescent protein (YFP) was detected in *Nicotiana benthamiana* epidermal cells coexpressing CsAIL6-cYFP and CsAN11-nYFP (Fig. 5b). These results indicated that CsAIL6 can directly interact with CsAN11 rather than CsAN1 or CsPH4 and negatively regulate its transcript level.

### Identification and functional confirmation of CsAN11

**Figure 6:**
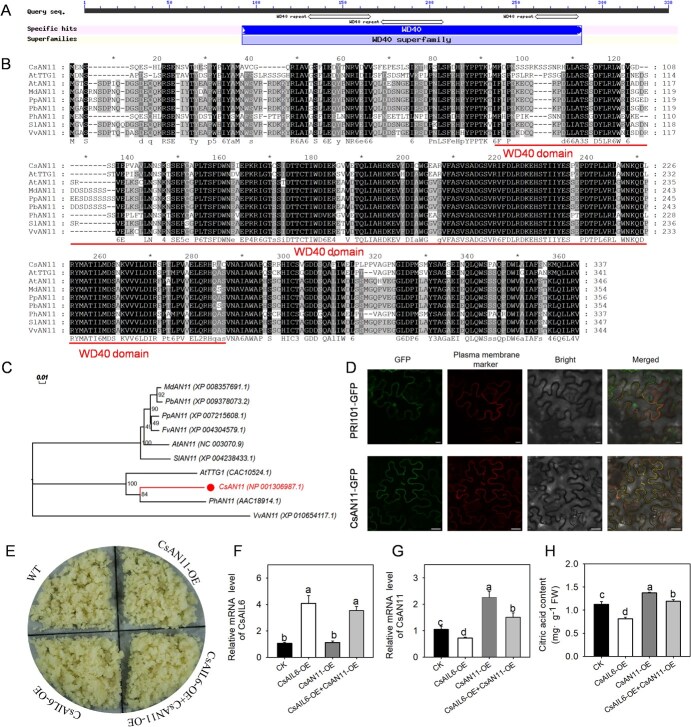
Identification, subcellular localization, and functional analysis of CsAN11. **A)** The putative conserved domains of CsAN11. **B)** Multiple protein sequence alignment of CsAN11 and its homologous sequences. At, *Arabidopsis thaliana*; Md, *Malus domestica*; Pb, *Pyrus bretschneideri*; Pp, *Pyrus persica*; Ph, *Petunia hybrida*; Sl, *Solanum lycopersicum*; Vv, *Vitis vinifera*; Cs, *Citrus sinensis*. **C)** Phylogenetic tree from different homologous sequences of CsAN11. CsAN11 was highlighted with the solid circle. The phylogenetic tree was constructed using the NJ bootstrap method (1000 replicates) after sequence alignment with MEGA-X (v10.2.2) software. **D)** Subcellular localization of the CsAN11–GFP fusion protein in the epidermal cells of *N. benthamiana* leaves. Scale bar = 25 μm. **E)** Calli phenotypes of WT, CsAIL6, and CsAN11 single and cotransferable calli (overexpression). **F–G)** Expression levels of CsAIL6 **F)** and CsAN11 **G)** in CsAIL6 and CsAN11 single and cotransferable calli. **H)** Citric acid contents in CsAIL6 and CsAN11 single and cotransferable calli. Different lowercase letters indicate statistically significant differences by the Duncan test (*P* < 0.05). Error bars indicate the standard deviation of three replicates (±SD). FW, fresh weight. WT, wild-type.

To doubtlessly elucidate the mechanism for CsAIL6 regulating citric acid accumulation in citrus fruits, *CsAN11* was then identified. Its coding sequence length is 1014 bp and encodes a protein of 337 amino acids. The putative conserved domains and sequence alignment revealed that the predicted protein contained three conserved WD40 repeat regions (Fig. 6a), and the amino acid sequences were highly similar to those of its orthologous species (Fig. 6b). Phylogenetic analysis indicated that CsAN11 was closely related to petunia PhAN11 (Fig. 6c). Subcellular localization analysis revealed that the CsAN11-GFP fluorescence signal was detected only on the plasma membrane (Fig. 6d).

Subsequently, *CsAIL6* and *CsAN11* overexpression vectors were transformed into citrus calli (Fig. 6e). Compared with those in the control calli, the transcript levels of *CsAIL6* and *CsAN11* in the *CsAIL6*-OE calli were significantly increased and decreased, respectively (Fig. 6f and g), whereas the citric acid content in the *CsAIL6*-OE calli was significantly decreased (Fig. 6h). In the *CsAN11*-OE calli, the *CsAIL6* expression level was similar as in the control calli (Fig. 6f); on the other hand, the *CsAN11* expression level (Fig. 6g) and the citric acid content (Fig. 6h) were obviously increased, compared with those in the WT lines. Moreover, when *CsAIL6* and *CsAN11* were cotransformed into calli, the transcript levels of *CsAIL6* and *CsAN11* were markedly higher than those in the WT calli (Fig. 6f and g); in addition, the citric acid content was distinctly higher than that in the *CsAIL6*-OE calli and was still significantly lower than that in the *CsAN11*-OE calli (Fig. 6h). Therefore, CsAN11 functions downstream of *CsAIL6* to regulate citric acid accumulation.

## Discussion

As a primary metabolite, citric acid content contributes greatly to citrus fruit flavor and market acceptability [5, 43]. Therefore, exploring citric acid accumulation mechanism becomes one of the research hotspots in order to improve fruit quality and ultimately enhance citrus industry growth [4]. It is clear that citric acid level varies among citrus varieties and at different developmental stages within the same variety (Fig. 2). The use of citrus varieties with a uniform genetic background and special citric acid traits is an important and effective strategy to study the mechanism of citric acid accumulation [30, 44]. HAL is a low-acid bud mutant of AL while WSY and HB are two pumelo cultivars with large differences in citric acid content [29]. Through integrated systems biology analysis of the transcriptomes, Huang *et al.* [23] found that *CsAIL6,* belonging to an AP2 transcription factor gene [45], was possibly involved in the regulation of citrus fruit acidity because it was differently expressed among fruits of four sweet orange varieties that differed in fruit acidity at two different developmental stages (cell division and cell expansion). In this study, we comparatively analyzed the dynamics of *CsAIL6* expression profiles and citric acid content during fruit development of four citrus cultivars (AL, HAL, HB, and WSY). Results showed that CsAIL6 expression levels in HAL and WSY fruits (with low citric acid content) were significantly higher than those in AL and HB fruits (with high citric acid content) at different stages of fruit development (Fig. 3b), which is consistent with the results of Huang *et al.* [23] Moreover, investigation of 18 citrus cultivars with different acidity indicated that the *CsAIL6* expression level was significantly and positively correlated with pH (r_1_ = 0.881) or significantly and negatively correlated with the TA content (r_2_ = −0.542) (Fig. 2a). Hence, this study further confirmed that *CsAIL6* negatively regulates citric acid accumulation (Figs. 2 and 3).

Citric acid accumulation is directly regulated by biosynthesis-, degradation-, and transport-related genes [2, 4]. To date, many reports suggested that the P-type proton pump gene (*CitPH5* or *CsPH8*) is the key factor regulating citric acid storage in cell vacuoles [28, 30, 31, 39]. Also, plenty of studies found that the P-type proton pump gene was regulated by various transcription factors such as MYB and bHLH transcription factors [31–35]. AP2/ERF transcription factors are a large plant-specific superfamily with five subfamilies (DREB, ERF, AP2, RAV, and the soloist superfamily) with different functions in regulating the stress tolerance and secondary metabolite biosynthesis [46–48]. Recent studies indicated that two citrus ERF transcription factors, CitERF13 and CitERF6, regulate organic acid accumulation through interacting with CitVHA-c4 and CitAclα1, respectively [49, 50]. Moreover, Zheng *et al.* [42] identified an apple AP2/ERF transcription factor, MdESE3, which can control fruit acidity through directly binding the promoter region of apple p-type proton pump gene (*MdMa11*). Here, we found that CsAIL6 contains two typical AP2 DNA-binding domains (Fig. 1b) and belongs to an AP2 transcription factor. We also proved that CsAIL6 has no transcriptional activation activity (Fig. S1), which means that it may not directly bind the promoter of structural genes such as *CsPH8* but can interact with other proteins to control fruit acidity.

To investigate the mechanism for CsAIL6 regulating citric acid accumulation in citrus fruits, we subsequently analyzed its interaction with proteins involved in the control of the P-type proton pump gene. To date, previous studies proved that the MBW complex (PH4–AN1–AN11) directly regulates the transcript level of the P-type ATPase gene [31–36, 39]. Some transcription factors, such as CitTRL, CsABF3, or AN2 (Ruby1/MYB), were found to directly interact with citrus AN1 to influence citric acid accumulation [5, 36, 39]. To investigate how CsAIL6 regulates citric acid accumulation, we compared the transcript levels of genes involved in citric acid metabolism and transport between the transgenic and control lines. We found that silencing *CsAIL6* significantly increased the transcript levels of *CsAN11*, *CsAN1*, and *CsPH4*, whereas overexpressing *CsAIL6* just significantly decreased only the *CsAN11* transcript level; moreover, only the tomato *AN11* (*SIAN11*) transcript level was significantly reduced in *CsAIL6*-OE tomato lines (Fig. 4). In addition, we also conducted Y2H and BIFC experiments and found that CsAIL6 only interacts with CsAN11 rather than with CsAN1 or CsPH4 (Fig. 5). These results supported that CsAIL6 participates in regulating citric acid accumulation in citrus fruits by interacting with CsAN11 rather than with CsAN1 or CsPH4.

AN11 is a WD40 protein that contains a signature WD (Trp-Asp) dipeptide and 40 amino acids in single repeats [51]. Although previous studies have confirmed the AN11 role in regulating vacuolar acidification and anthocyanin synthesis by interacting with MYB and bHLH transcription factors to form the MBW complex [8, 33, 52], the function of citrus AN11 gene (*CsAN11*) was rarely reported before in citrus species. In this study, we further identified CsAN11 and found that it contains a WD domain and belongs to the WD40 protein; moreover, overexpressing *CsAN11* did significantly increase the citric acid content in citrus calli (Fig. 6). These results indicated that the citrus AN11 is a WD40 protein and has a function similar to that of PhAN11 for vacuolar acidification [33].

Taken together, the present study identified an AP2 transcription factor, CsAIL6; it negatively regulates citric acid accumulation in citrus fruit by interacting with the WD40 protein CsAN11 rather than with CsAN1 or CsPH4 of the MBW complex. This study presents a novel regulatory mechanism of AP2–WD40 for citric acid accumulation, which improves the knowledge about the regulation of vacuolar acidification and provides another choice for fruit quality improvement.

## Materials and methods

### Plant materials and treatment

The 18 citrus accessions from different cultivars were grown and conventionally managed in Huazhong Agricultural University (Wuhan, China) (Table S1). They were subsequently grafted onto trifoliate orange (*Poncirus trifoliate*). At fruit harvest, five healthy fruits of comparable size were randomly collected from each tree in various directions at the periphery of the canopy; three biological replicates were conducted for each cultivar, and one normal-growing tree was considered a replicate. A portion of each sample was used directly for pH and TA determination. The fruit juice sacs of the other samples were separated, rapidly frozen by liquid nitrogen and stored at −80°C for following use.

Moreover, the fruits of four citrus cultivars ‘Anliu’ sweet orange (AL, *Citrus sinensis* cv. Anliu), ‘Hong Anliu’ sweet orange (HAL, *C. sinensis* cv. Honganliu), ‘HB’ pomelo (*Citrus grandis* cv. Huayou No.1) and ‘Wusuan’ pomelo (WS, *C. grandis* cv. Wusuan), at different development stages were collected for citric acid and RNA extraction. Specifically, ‘AL’ and ‘HAL’ fruits were collected at 100, 130, 160, 190, and 220 DAFB, and ‘HB’ and ‘WS’ fruits were collected at 105, 145, 175, and 190 DAFB. Fruits were harvested and further processed for citric acid measurement and RNA extraction, as mentioned above.

In addition, fibrous roots, fully blooming flowers, mature leaves, young leaves, and ripening fruits (juice sacs) were collected from ‘Newhall’ navel orange plants for tissue-specific expression analysis. The ‘Micro-Tom’ (*Solanum lycopersicum*) cultivar was selected as the genetic transformation material for CsAIL6 function analyses, and *N. benthamiana* was used for subcellular localization and BIFC analysis. ‘Micro-Tom’ and *N. benthamiana* were planted in a climate chamber (16 h light/8 h dark, 25°C).

### Bioinformatics and phylogenetic analysis

The sequences of CsAIL6 and CsAN11 were downloaded from the Citrus Pan-genome to Breeding Database (http://citrus.hzau.edu.cn/index.php). The gene intron/exon structure was analyzed using the Gene Structure Display Server (GSDS, http://gsds.cbi.pku.edu.cn/) [53]. The conserved domains were predicted in CCD (https://www.ncbi.nlm.nih.gov/cdd). Homologous CsAIL6 proteins were identified from other species based on their deduced proteins in the National Center for Biotechnology Information (NCBI), after which protein sequences with high similarity across each species were downloaded from NCBI. The primary protein sequence was predicted using the online ExPASy Bioinformatic Resource Portal (https://web.expasy.org/cgi-bin/protparam/protparam). Sequence alignment was carried using the ClustalW method with MEGA-X. The alignment results were used to construct the phylogenetic tree with the neighbor-joining (NJ) method.

### Determination of titratable acid and citric acid contents

TA was measured by titration with sodium hydroxide (NaOH, 0.1 M) and phenolphthalein (1%) as pH indicators [54]. The citric acid content was determined by gas–liquid chromatography as described in a previous study [55].

### RNA extraction and quantitative real-time PCR

Total RNA of the fruit samples was extracted as mentioned previously [56]. The *TransScript* One-step gDNA Removal and cDNA Synthesis SuperMix (TransGen Biotech, Beijing, China) was used to synthesize cDNA from 1 μg of high-quality total RNA. The qRT-PCR was performed in a 10-μl reaction volume by using Hieff qPCR SYBR Green Master Mix (YEASEN, Shanghai, China) in the QuantStudio™ 6 Flex Real-Time PCR System (Thermo Fisher Scientific, USA), following the manufacturer’s protocol. The qRT-PCRs were normalized to actin gene Ct values as previously mentioned [57, 58]. All data were analyzed by using the Livak method [59]. All primers are listed in Supplemental Table S2.

### Subcellular localization analysis

For the subcellular localization assay of CsAIL6, the CDSs without termination codons of *CsAIL6* and *CsAN11* were inserted into the pRI101-GFP vector, respectively. The specific primers are listed in Supplemental Table S2. The fusion constructs were subsequently transformed into the chemically competent cell EHA105. The fusion constructs and markers were transiently cotransformed into *N. benthamiana* leaves as mentioned previously [60]. After 2 days of infiltration, the subcellular localization was visualized under a confocal laser scanning microscope (Leica SP8, Wetzlar, Germany).

### Construction of expression vectors and gene transformation

To construct the OE vectors, the CDSs of *CsAIL6* and *CsAN11* were amplified from ‘AL’ sweet orange and inserted into the binary overexpression vector pK7WG2D using gateway technology, respectively. A 300-bp reverse complement fragment from *CsAIL6* was cloned and inserted into the pTRV2 vector to construct VIGS vectors. All the fusion constructs were individually introduced into the chemically competent cell EHA105. The *CsAIL6*-OE fusion vector was subsequently transformed into tomato mediated by *Agrobacterium tumefaciens* using the leaf disc infiltration method [61].

The transient transformation of OE and VIGS were conducted in the fruit segments of citrus cv. ‘Nanfeng’ at 150 DAFB, following a previously described method with minor modifications [62]. The *Agrobacterium* strains expressing *CsAIL6*-OE, the empty vector pK7WG2D, pTRV2-CsAIL6, and pTRV1 were cultured in LB liquid medium for 12 h. The OD_600_ values of the *Agrobacterium* suspensions were adjusted to 0.8–1.0 with buffers prepared with 10 mM MgCl_2_, 10 mM MES (pH -5.6), and 200 μM AS. In addition, the resuspensions of pTRV1 and pTRV2-CsAIL6 were mixed at a 1:1 ratio to generate *Agrobacterium* infestation solution. Five to ten uniform healthy fruits located inside the canopy were selected and injected with *Agrobacterium* carrying the empty vector on one side of the same fruit, while *Agrobacterium* containing the expression vector of the target gene was injected on the opposite side. The 200 μl *Agrobacterium* solution was gently injected into the epicarp at a depth of 0.1–0.3 cm using a 1-ml syringe. At 5 days after injection, the injected fruit juice sacs were collected for citric acid determination and gene expression analysis, and each fruit was considered a replicate.

The *Agrobacterium*-mediated transient genetic transformation of citrus calli was performed following the previous method with slight modifications [63]. The ‘Valencia’ orange calli used for genetic transformation were subcultured on MT medium at 25°C. The citrus calli were precultured in MT liquid medium at a constant temperature (21°C) on a shaker at 110 rpm for 3 days. The activated *Agrobacterium* was suspended in MT liquid medium in a shaker at 180 rpm for ~2 h until the OD_600_ was between 0.6 and 0.8. The precultured calli were immersed in infiltration solution and shaken for 30 min, after which their surfaces were blotted dry with sterile filter paper, and transferred to coculture medium in the dark at 21°C. Five days after infection, the samples were collected, snap-frozen in liquid nitrogen, and stored at −80°C for further analysis of the citric acid content and gene transcription levels. The experiment was repeated with at least three biological replicates. The positive plants were identified by PCR amplification of genomic DNA and qRT-PCR using the primers are listed in Supplemental Table S2.

### Yeast two-hybrid assay

The CDS of *CsAIL6* was amplified and introduced into the pGBKT7 (BD) vector, and *CsAN11*, *CsAN1*, and *CsPH4* were individually cloned and introduced into the pGADT7 (AD) vector. The Y2H assay was conducted as mentioned previously [64]. All primers used for Y2H assay are listed in Supplemental Table S2.

### Bimolecular fluorescence complementation

The BIFC experiments were conducted as previously described [65]. The CDSs of *CsAIL6* and *CsAN11* without stop codons were inserted into the C-terminal (YCE) and N-terminal (YNE) of the YFP-101 vector, respectively. These fusion constructs were subsequently transformed into the chemically competent cell EHA105, and then coinjected into the 5-week-old *N. benthamiana* leaves with equal volumes of strains containing different fusion vectors. The YFP fluorescence signal was detected 2 days after transfection under the confocal laser scanning microscope. The specific primers are listed in Supplemental Table S2.

### Statistical analysis

All the data were analyzed by applying one-way ANOVA or *t*-test of IBM SPSS Statistics v.26, and the level of significance was set at *P* < 0.05.

## Accession Numbers

Sequence data from this study can be found in Citrus Pan-genome to Breeding Database under accession numbers: *CsAIL6* (Cs_ont_1g022280.1), *CsAN11* (Cs9g04820.1), *CsAN1* (Cs5g31400.2), *CsPH4* (Cs9g03070.1).

## Supplementary Material

Web_Material_uhaf002

## Data Availability

All data supporting the findings of this study are available within the article and its supplementary materials published online, and they are available upon reasonable request.
