# Supplementary material for: AP2 transcription factor CsAIL6 negatively regulates citric acid accumulation in citrus fruits by interacting with a WD40 protein CsAN11
Source: Hortic Res. 2025 Jan 6;12(4):uhaf002. doi: 10.1093/hr/uhaf002 (PMC11896974; doi:10.1093/hr/uhaf002)
Supplement: Web_Material_uhaf002 [file web_material_uhaf002.zip › Supplementary materials.docx]

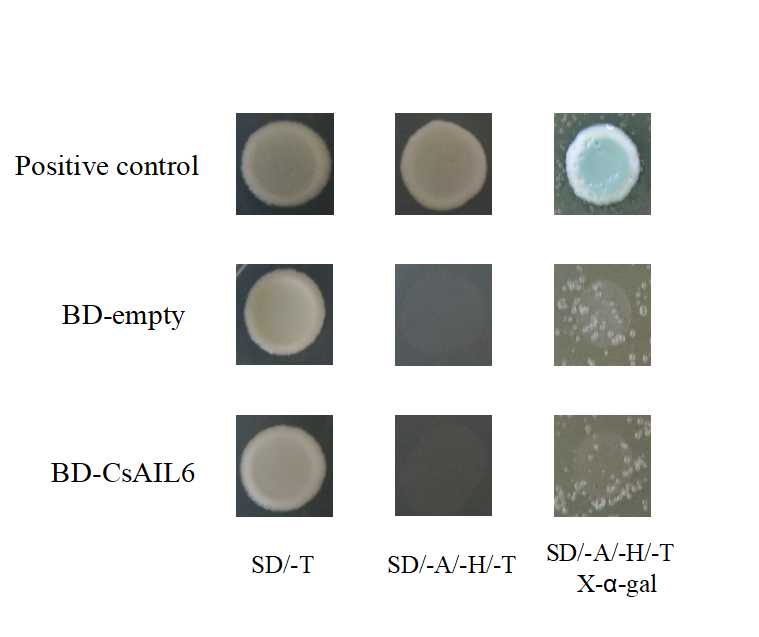


**Figure S1.** Transcriptional activation analysis of CsAIL6. BD-empty indicates pGBKT7; BD-CsAIL6 indicates pGBKT7-CsAIL6. The pGBKT7-p53 and pGBKT7-empty vectors were used as positive and negative controls.

**Table S1.** Basic information of 18 fruit samples harvested on Nov. 10, 2022

| **Sample name** | **Citrus cultivar** | **pH in fruit juice sacs** | **TA (%) in fruit juice sacs** |
| --- | --- | --- | --- |
| AL | ‘Anliu’ orange | 3.67 | 0.65 |
| HAL | ‘Honganliu’ orange | 5.77 | 0.13 |
| BTC | ‘Bingtang’ orang | 3.94 | 0.54 |
| DHT | ‘Dahongtian’ orange | 2.94 | 1.28 |
| NHE | ‘Newhall’ navel orange | 3.67 | 0.65 |
| AY | ‘Anyuan’ navel orange | 3.97 | 0.54 |
| NF | ‘Nanfengmiju’ tangerine | 3.48 | 0.77 |
| WM | ‘Guoqing No.1’ mandarin | 3.58 | 0.61 |
| PG | ‘Huagan No.2’ Ponkan | 3.16 | 0.96 |
| BZH | ‘Shiranui’ tangerine | 3.62 | 0.60 |
| WG | ‘Orah’ mandarin | 2.94 | 1.31 |
| MKT | ‘W. Murcott’ | 3.02 | 1.27 |
| CX | ‘Haruka’ | 3.47 | 0.73 |
| NM | ‘Eureka’ lemon | 2.30 | 6.01 |
| TNM | Sweet lemon | 5.76 | 0.12 |
| HB | ‘HB’ pomelo | 3.08 | 1.29 |
| WS | ‘Wusuan’ pomelo | 5.86 | 0.09 |
| MJY | ‘Majia’ pomelo | 3.16 | 1.17 |

**Table S2.** Primers and probes used in this study

| 1. Primers used for plant transformation vector | |
| --- | --- |
| *CsAIL6*-pK7 | F: ggggacaagtttgtacaaaaaagcaggctccATGGCTCCGGCGAGTAACTG |
|  | R: ggggaccactttgtacaagaaagctgggttTCAATAGGATTGGTGAGGCCA |
| *CsAN11*-pK7 | F: ggggacaagtttgtacaaaaaagcaggctccATGGAAAACTCAAGCCAAGAATCC |
|  | R: ggggaccactttgtacaagaaagctgggttTCAAACTTTCAAAAGTTGCATTTTG |
| *CsAIL6*-TRV2 | F: gtgagtaaggttaccgaattcATGTGGTCGTCATCATCATC |
|  | R: cgtgagctcggtaccggatccTTCAGTTTGGCTATGAACAAGAGATG |
| 2. Primers used for the positive identification | |
| 35S | F: TGACGCACAATCCCACTATCCTTCG |
| CsAIL6 | R: TCAATAGGATTGGTGAGGCCA |
| 3. Primers used for subcellular localization, Y2H and BiFC | |
| CsAIL6-PRI101 | F: catatgcccgtcgaccccgggATGGCTCCGGCGAGTAACTG |
|  | R: gctcaccatgaattcggatccATAGGATTGGTGAGGCCATAAGAA |
| CsAN11-PRI101 | F: tcttcactgttgatacatatgATGGAAAACTCAAGCCAAGAATCC |
|  | R: ttcggatccggtacccccgggAACTTTCAAAAGTTGCATTTTGTTAGA |
| CsAIL6-BD | F: aggccgaattcccggggatccATGGCTCCGGCGAGTAACTG |
|  | R: ggttatgctagttatgcggccgcTCAATAGGATTGGTGAGGCCA |
| CsAN11-AD | F: gtaccagattacgctcatatgATGGAAAACTCAAGCCAAGAATCC |
|  | R: cagctcgagctcgatggatccTCAAACTTTCAAAAGTTGCATTTTG |
| CsAN1-AD | F: gtaccagattacgctcatatgATGGATGCTCCGCCGCCG |
|  | R: cagctcgagctcgatggatccTTAATTGACATACTGGGGTATTATTTGA |
| CsPH4-AD | F: gtaccagattacgctcatatgATGAGGAACCCATCAACATCACC |
|  | R: ccgtatcgatgcccacccgggCTACTCAACGTGTTCATCAACCCT |
| CsAIL6-nYFP | F: agtgaattcatcgatggatccATGGCTCCGGCGAGTAACTG |
|  | R: caacttttgctccatcccgggATAGGATTGGTGAGGCCATAAGAA |
| CsN11-cYFP | F: agtgaattcatcgatggatccATGGAAAACTCAAGCCAAGAATCC |
|  | R: atcgtatgggtacatcccgggAACTTTCAAAAGTTGCATTTTGTTAGA |
| 4. Primers used for qRT-PCR analysis | |
| *CsAIL6* | F: CGGCGAGTAACTGGCTTTCA |
|  | R: TGTCCAGCCAGCATTAGCAT |
| *CsActin* | F: CCGACCGTATGAGCAAGGAAA |
|  | R: TTCCTGTGGACAATGGATGGA |
| *SlActin* | F: CCTCAGCACATTCCAGCAG |
|  | R: CCACCAAACTTCTCCATCCC |
| *CsAN11* | F: GCTGTTGATTGAAGTTACTG |
|  | R: CATTAGCTTGGTGGGTGGGT |
| *CsAN1* | F: GATCCGAGAACTCCGTGACC |
|  | R: CCTCTTAACCTCCACAATG |
| *CsPH4* | F: ACGTGTCCGACATGAAATTG |
|  | R: AGATGATGAAGTGGAACAAGTGC |
| *SlAN11* | F: AACACCCTTATCCACCAA |
|  | R: TCTCGGCTCCACTTCATT |
| *SlAN1* | F: GGAAGTATAGTCGGAGCA |
|  | R: TTACATCTAACAACAACCCT |
| *SlPH4* | F: TCCTGGAGAAACTACGAG |
|  | R: GTGAAGATGAAGCCATAAAA |
